# Graphene-Based Long-Period Fiber Grating Surface Plasmon Resonance Sensor for High-Sensitivity Gas Sensing

**DOI:** 10.3390/s17010002

**Published:** 2016-12-22

**Authors:** Wei Wei, Jinpeng Nong, Guiwen Zhang, Linlong Tang, Xiao Jiang, Na Chen, Suqin Luo, Guilian Lan, Yong Zhu

**Affiliations:** 1 Key Laboratory of Optoelectronic Technology & Systems, Ministry of Education of China, College of Optoelectronic Engineering, Chongqing University, Chongqing 400044, China; nongjp@cigit.ac.cn (J.N.); 20093440@cqu.edu.cn (G.Z.); 20103279@cqu.edu.cn (X.J.); 20150802007@cqu.edu.cn (N.C.); 20133092@cqu.edu.cn (G.L.); 2 Chongqing Research Center for Advanced Materials, Chongqing Academy of Science and Technology, Chongqing 401123, China; ssuqinluo@163.com; 3 Chongqing Institute of Green and Intelligent Technology, Chinese Academy of Sciences, Chongqing 401122, China; tll@cigit.ac.cn; 4 Chongqing Engineering Research Center of Graphene Film Manufacturing, Chongqing 401329, China

**Keywords:** surface plasmon resonance, graphene, long-period fiber grating, sensitivity

## Abstract

A graphene-based long-period fiber grating (LPFG) surface plasmon resonance (SPR) sensor is proposed. A monolayer of graphene is coated onto the Ag film surface of the LPFG SPR sensor, which increases the intensity of the evanescent field on the surface of the fiber and thereby enhances the interaction between the SPR wave and molecules. Such features significantly improve the sensitivity of the sensor. The experimental results demonstrate that the sensitivity of the graphene-based LPFG SPR sensor can reach 0.344 nm%^−1^ for methane, which is improved 2.96 and 1.31 times with respect to the traditional LPFG sensor and Ag-coated LPFG SPR sensor, respectively. Meanwhile, the graphene-based LPFG SPR sensor exhibits excellent response characteristics and repeatability. Such a SPR sensing scheme offers a promising platform to achieve high sensitivity for gas-sensing applications.

## 1. Introduction

The fiber optic surface plasmon resonance (SPR) sensor [1,2,3] has been widely used in biochemistry, medical analysis and environmental monitoring, owing to its fast response, being label-free, and its real-time detection. In order to improve the sensitivity of the sensor, various fiber SPR structures have been designed to effectively couple the fiber core mode into the SPR mode on the fiber surface. The most straightforward approach is utilizing the geometry-modified fiber by removing the cladding entirely or partially, such as etched fibers [4], side-polished fibers [5], tapered fibers [6], U-shaped fibers [7], and D-shaped fibers [8]. However, the circular symmetry of the fibers is inevitably broken, resulting in polarization dependence [9]. Meanwhile, the asymmetric destruction of the modified fibers weakens the mechanical stability of the fiber optic SPR sensors. 

To overcome these drawbacks, the fiber grating–assisted SPR sensors are designed by integrating metal overlays on the fiber gratings, which exhibits advantages over the modified fiber-based SPR sensors, such as preservation of the structural integrity and circular symmetry of the fiber [9]. These periodic grating structures inscribed in the fiber core by a laser offer a new strategy to improve the conversion efficiency of the fiber core mode into the SPR mode. Typically, fiber Bragg grating (FBG) and long-period fiber grating (LPFG) are two kinds of grating-assisted SPR platforms that are often used. Compared to the FBG (a grating period of a few hundred nanometers) [10,11], LPFG has a longer grating period (a few hundred microns) [12,13], which can couple more energy of the fiber core guide mode into the co-propagating cladding mode, and hence further improve the excitation efficiency of the SPR [9]. It has been widely reported that LPFG SPR sensors possess impressive detection sensitivity [9,14,15]. However, further improvement of the sensitivity is essentially limited due to the fact that the metals cannot effectively adsorb the molecules because of their high surface inertness and intrinsic hydrophobic. 

Compared to the metals, graphene [16,17,18,19,20], which emerged as a revolutionary two-dimensional material, possesses extraordinary physical and chemical properties, and it offers a unique opportunity to address this situation. Its high surface-to-volume ratio and good biocompatibility allow the effective adsorption of molecules on its surface [21]. These excellent features make graphene a highly promising candidate as the sensing layer for various fiber optic sensors [22], such as graphene-covered tapered multimode fibers [23], graphene-based D-shaped fibers [24], and graphene-coated microfiber Bragg grating [25]. Motivated by these ideas, we consider the combination of graphene with the LPFG SPR sensor, where a layer of graphene is covered onto the Ag film surface of the LPFG SPR sensor. The presence of the graphene enhances the intensity of the evanescent field on the surface of the fiber and thereby the interaction between the SPR wave and molecules, which would significantly improve the sensitivity of the LPFG SPR sensor and may provide a new opportunity for the development of fiber optic SPR sensors. An experimental setup is built to evaluate the sensitivity of the graphene-based LPFG SPR sensor, as well as the response characteristics and repeatability.

## 2. Principles and Experimental Setup 

**Figure 1:**
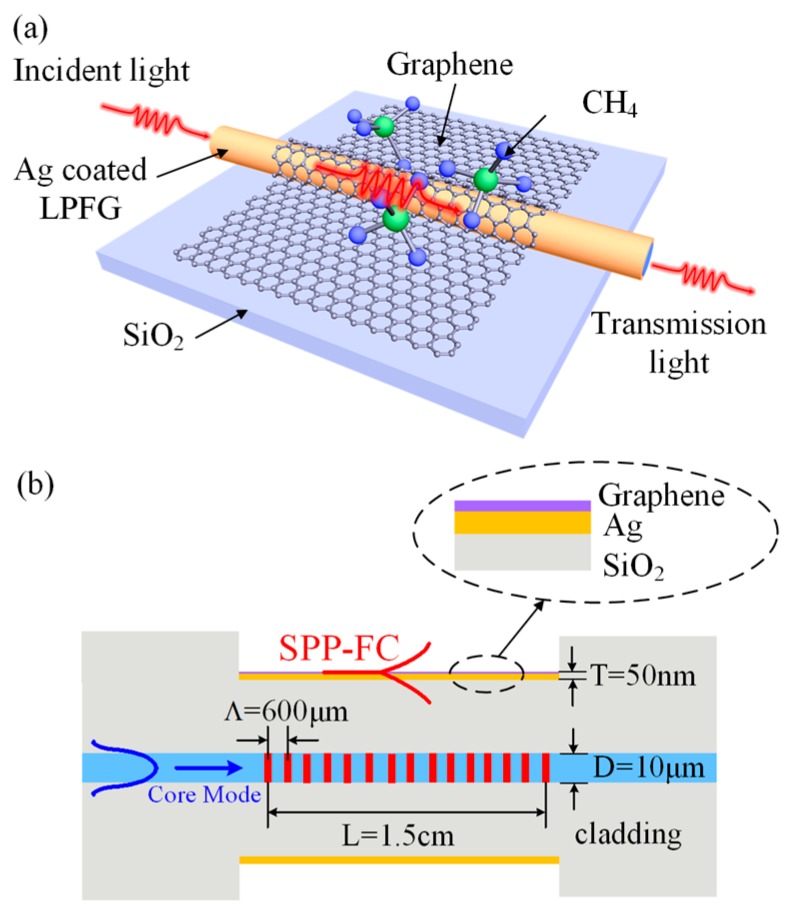
(**a**) Schematic of the graphene-based LPFG SPR sensor; (**b**) Longitudinal section of the graphene-based LPFG SPR sensor.

The structure of the proposed graphene-based LPFG SPR sensor is schematically shown in Figure 1a. The LPFG is first inscribed on the fiber core by a CO_2_ laser. Then the LPFG SPR sensor is fabricated by coating Ag film on the LPFG surface and fixing it on a SiO_2_ substrate. Finally, the chemical vapor deposition (CVD)-grown graphene monolayer is covered onto the Ag film surface of the LPFG SPR sensor to form the graphene based LPFG SPR sensor. Its longitudinal section is shown in Figure 1b. The period *Λ* and the length *L* of the LPFG are 600 μm and 1.5 cm, the diameter of the corroded fiber is 100 μm, the diameter *D* of the fiber core is 10 μm, and the thickness *T* of the Ag film is 50 nm.

As illustrated in Figure 1b, the forward-propagating fundamental (core) mode of the optical fiber is reflected by the LPFG and coupled into the co-propagating cladding modes. When the axial component of the propagation constant of the cladding mode matches that of the SPR, coupling between the SPR mode and the cladding mode occurs, resulting in a mixed surface plasmon resonance–fiber cladding (SPR-FC) hybrid mode [11]. Therefore, the transfer of electromagnetic wave energy between the hybrid mode and cladding mode can occur once the propagation constant of the fundamental mode diffraction altered by the LPFG is equal to the propagation constant of the SPR-FC mode. This coupling condition is fulfilled at the resonance wavelength which can be expressed as [11]:

(1) $$\lambda_{res}^{i}=ℜ(n_{eff}^{1}+n_{eff}^{i})\Lambda$$

where $\lambda_{res}^{i}$ represents the resonance wavelength corresponding to the *i*th cladding mode, ℜ represents the real part, *Λ* is the grating period, $n_{eff}^{1}$ and $n_{eff}^{i}$ are the effective refractive index of the fundamental core mode and the *i*th SPR-FC modes, respectively. 

Once the light is coupled into the SPR-FC hybrid modes, it will decay immediately due to the scattering losses, which provides attenuation bands at the resonant wavelength in the transmission spectrum. Due to coupling with the SPR, the propagating constant of this hybrid mode is highly sensitive to the refractive index changes of the analyte. A change in the external refractive index induced by the variation of the gas concentration leads to the change in the propagating constant of the hybrid mode, thereby resulting in the variation of the resonant wavelength. Then the change of the refractive index can be measured by detecting the variation of the resonant wavelength. The sensitivity *S* of the sensor is defined as the ratio of the shift in the resonant wavelength to the change in the external refractive index *n_sur_* expressed as:

(2) $$S=\frac{d\lambda_{res}^{i}}{dn_{sur}}=\Lambda \frac{dR\left\{n_{eff}^{i}(\lambda_{res}^{i})\right\}}{dn_{sur}}$$

This equation indicates that the sensitivity of the sensor is proportional to the effective refractive index of the *i*th SPR-FC modes and the grating period of the LPFG. When the LPFG SPR sensor is further coated with a graphene layer, it is expected to increase the intensity of the SPR-FC modes at the fiber/metal interface and consequently improve the sensitivity of the sensor.

## 3. Experiment Section

### 3.1. Fabrication of the Graphene-Based LPFG SPR Sensor

**Figure 2:**
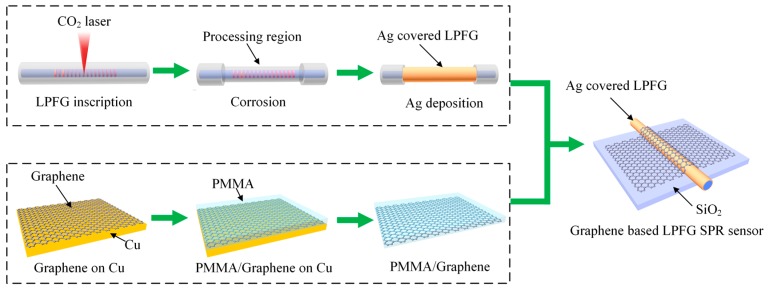
Fabrication process of the proposed graphene-based LPFG SPR sensor.

The fabrication process of the proposed graphene-based LPFG SPR sensor is schematically demonstrated in Figure 2. The LPFG inscription was performed according to the method previously reported [13], using a single mode fiber with core diameter of 10 μm, cladding diameter of 125 μm, and numerical aperture of 0.22. Briefly, the fiber (Corning SMF-28, Shanghai, China) was scanned by means of a 2D optical scanner attached to a CO_2_ laser (10 W full power, 10 kHz frequency, Synrad Inc., Mukilteo, WA, USA) with a computer control at a speed of 2.4 mm/s. The laser beam was focused to a spot with a diameter of ~50 μm. In our work, the length of the processing area is 1.5 cm with grating period *Λ* of around 600 μm. The LFBG processing area was then corroded to 100 μm in hydrofluoric acid for 12 min, followed by the thermal evaporation of 50 nm Ag film. The thickness of the depositing silver film on the fiber core can be controlled and monitored in real time, which is measured by a quartz crystal monitor in the thermal evaporation machine.

Then, the large-area graphene monolayer was synthesized by chemical vapor deposition (CVD) method on a high purity copper foil (No. 13439, Alfa Aesar, Shanghai, China), with flowing mixture of 3 sccm H_2_ (70 mTorr) and 30 sccm CH_4_ (340 mTorr) gases at 1000 °C. A layer of PMMA thin film was coated on as-grow graphene on Cu foil as a transfer supporting layer by a spin coater. The graphene on the other side was removed by oxygen plasma processing. After completely removing Cu by the solution of FeCl_3_ and HCl, the PMMA-supported floating graphene film was rinsed with deionized water twice and transferred onto the top of the fabricated sensor probe fixed on the SiO_2_ substrate. It was then directly covered around the Ag film surface of LPFG SPR sensor to form a graphene-based LPFG SPR sensor. Finally, the supporting PMMA layer were removed by acetone treatment.

### 3.2. Experimental Setup

**Figure 3:**
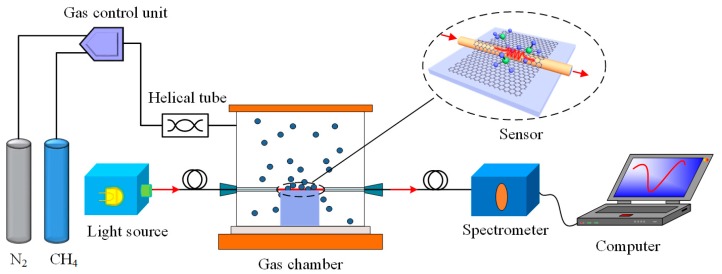
Scheme of sensing system based on the graphene/Ag-coated LPFG SPR sensor.

The experimental setup for the sensing system is schematically depicted in Figure 3, which includes two independent parts. The first part is the gas flow control system. Two mass flow controllers, individually controlled by an electronic unit, were employed to precisely control the flow of methane and nitrogen carrier gas into the gas chamber. A stainless steel helical tube was fixed to keep the gases well mixed. The second part of the setup is the optical system. The light source is a supercontinuum source (SC-5) (Wuhan Yangtze Soton Laser Co., Ltd., Wuhan, China) with wide spectral range from visible to near infrared region. The spectrum stability in the considering spectral range of 1500 nm to 1580 nm is better than 0.1% and the optical power is about 80 mW. The light emitted from the light source couples into the optical fiber and travels to the graphene based LPFG SPR sensor. The resonance wavelength shift induced by the change of the gas concentration in the chamber is captured by a fiber optic spectrometer (Agilent 86140B, San Jose, CA, USA) with a resolution of 0.01 nm and dark noise of 100 nW linked to a computer. In our experiment, the intensity of the light source is normalized and each concentration was measured at least five times. 

## 4. Results and Discussion

### 4.1. Characterizations

**Figure 4:**
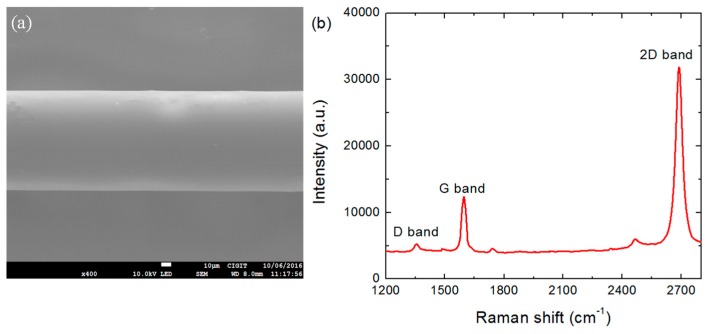
(**a**) SEM picture of the graphene-based LPFG SPR sensor; (**b**) Raman spectrum of monolayer graphene covered on the Ag-coated fiber surface.

The scanning electron microscopy images of the graphene-based LPFG SPR sensor are shown in Figure 4a. To evaluate the quality of the graphene, Raman spectra of graphene were recorded and are shown in Figure 4b. Obviously, three graphene characteristic peaks at 1353 cm^−1^ (D band), 1582 cm^−1^ (G band), and 2691 cm^−1^ (2D band) [26] can be observed, verifying the presence of graphene on the fiber surface. The peak of the G band at 1582 cm^−1^ corresponds to the graphitization of the sp^2^-bonded carbon atoms in the two-dimensional ordered hexagonal graphene. The peak of the 2D band at 2691 cm^−1^ is related to the inter-valley double-resonant Raman scattering. The high ratio of the peak intensities of the 2D band to the G band (ratio ≈ 2.5) indicates the Raman feature of the monolayer graphene. Meanwhile, the small peak of the D band near 1350 cm^−1^, corresponding to the disorderly carbon and defects, suggests the high quality of the graphene. 

### 4.2. Sensing Performance

**Figure 5:**
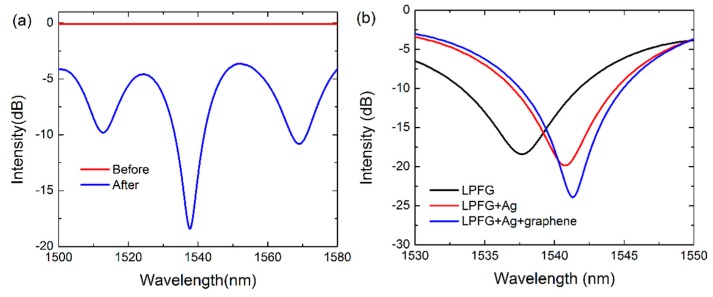
(**a**) Transmission spectra of the fiber before and after fabrication of LPFG; (**b**) The transmission spectra of LPFG sensor, Ag-coated LPFG SPR sensor and graphene-based LPFG SPR sensor.

To verify the successful inscription of the LPFG on the fiber core, the transmission spectra of the fiber before and after fabricating LPFG are provided in Figure 5a. Clearly, the transmission of the fiber before the fabrication of LPFG is almost zero since there is no refractive index modulation that occurs in the fiber core. After fabricating the LPFG by scanning CO_2_ laser pulses, three loss peaks can be observed at 1512.8 nm, 1537.7 nm and 1569.1 nm in the considered spectral window, indicating the successful fabrication of the LPFG on the fiber core. This is due to the conversion of the fundamental core mode to the co-propagating cladding modes with different diffraction orders, which leaks out from the fiber and interacts with the sensing medium. Meanwhile, the loss peak located at 1537.7 nm possesses the minimum value of transmission losses of −18 dB, which is deeper than that of the other two peaks. It means that the coupling efficiency of this loss peak is larger compared to the other two peaks, which provides a stronger interaction between the cladding mode and sensing medium, and thereby results in a higher sensitivity. Therefore, we mainly focus on analyzing the sensing performance of the sensors using this loss peak (1537.7 nm) in the following context.

We first investigate the spectral features of the three kinds of fabricated sensors, i.e., the LPFG sensor, the Ag-coated LPFG SPR sensor and the grapheme-based LPFG SPR sensor. It is found that the interaction between the incident light and the sensor can be enhanced by covering the Ag film and monolayer graphene onto the surface of the LPFG. The transmission spectra of these sensors are recorded and shown in Figure 5b. With respect to the LPFG sensor, the resonance wavelength of the Ag-coated LPFG SPR sensor shifts from 1537.7 nm to 1540.8 nm, along with the decrease of the minimum transmission loss from −18 dB to −20 dB. This is attributed to the excitation of the SPR mode induced by the Ag film which enhances the evanescent field intensity on the fiber surface. Moreover, compared to the Ag-coated LPFG SPR sensor, the resonance wavelength of the graphene-based LPFG SPR sensor further red-shifts to 1541.3 nm, and the minimum transmission loss reduces to −25 dB. These phenomena indicate that the presence of graphene can improve the conversion efficiency of the fiber core mode into the SPR mode.

**Figure 6:**
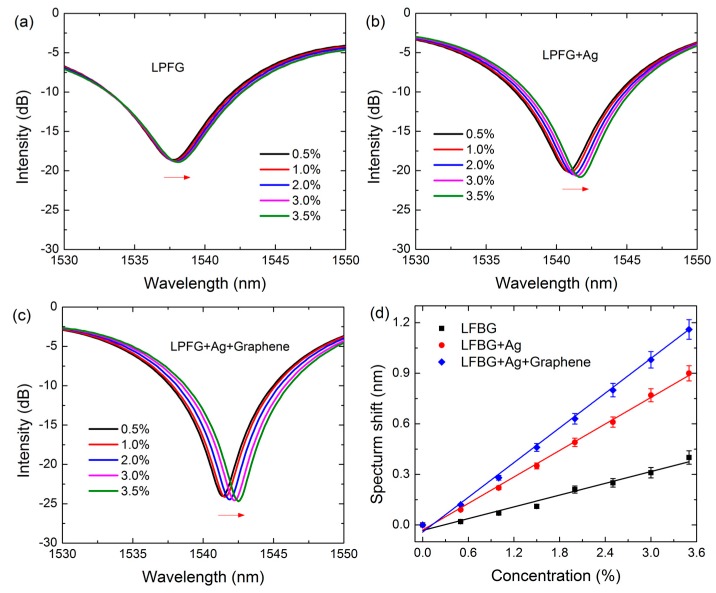
Transmission spectra of (**a**) LFBG sensor; (**b**) Ag-coated LFBG SPR sensor and (**c**) graphene-based LFBG SPR sensor with different concentrations of methane gas. (**d**) Resonance wavelength shift versus concentration of methane.

Then the sensitivities of the LPFG sensor, Ag-coated LPFG SPR sensor and graphene-based LPFG SPR sensor are evaluated by exposing the sensors directly to methane with various concentrations. The transmission spectra of the three kinds of sensors are shown in Figure 6a–c, respectively. One can see that the resonant spectra of the sensors all exhibit a wavelength red-shift with increasing methane concentrations. Specifically, the resonant wavelength shifts of the LPFG sensor, Ag-coated LPFG SPR sensor and graphene-based LPFG SPR sensor are 0.4 nm, 0.9 nm and 1.2 nm, respectively. This indicates that the graphene-based LPFG SPR sensor is the most sensitive to the change of the methane concentration, which further verified that the performance of the LPFG SPR sensor can be improved by the covering of graphene. To quantitatively compare the sensitivity of these three sensors, the resonance wavelengths shifts are extracted from Figure 6a–c as a function of the methane concentration, and plotted in Figure 6d. The linear fitted curves show that the plots of the calibration curves exhibit good linearity, with the correlation coefficients of 0.975, 0.995 and 0.995. The *y*-axis error bars indicate that the signals of the sensors are very stable. The calculated sensitivities of these sensors are 0.116 nm%^−1^, 0.262 nm%^−1^, and 0.34 nm%^−1^, with corresponding detection limits of 0.086%, 0.038%, and 0.029%, respectively, considering that the resolution of the optic spectrometer is 0.01 nm [27]; these values are comparable to or even better than that of the commercially available methane sensors (0.05%). This indicates that the graphene-based LPFG SPR sensor possesses the best sensing performance, the sensitivity of which is improved 2.96 and 1.31 times compared to the LPFG sensor and Ag-coated LPFG SPR sensor. 

### 4.3. Response Characteristics 

**Figure 7:**
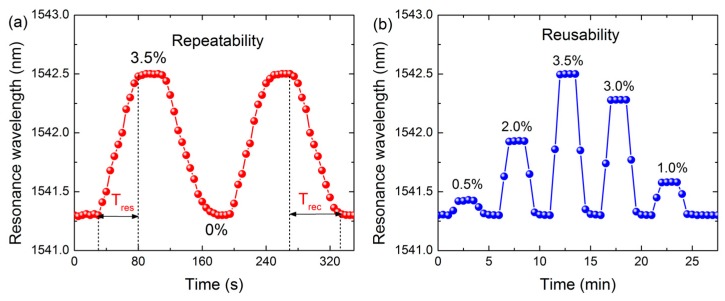
(**a**) Repeatability response curve of graphene-based LPFG SPR sensor to 3.5% methane gas sample. The signal was recorded every 5 s; (**b**) Reusability of graphene-based LPFG SPR sensor to methane gas sample with different concentrations. The signal was recorded every 30 s.

We further explore the response characteristics of the graphene-based LPFG SPR sensor. Experiments were performed successively exposing the sensor to a 3.5% methane concentration. The signal was collected every 5 s, and the extracted resonance wavelength versus the time is plotted in Figure 7a. It can be seen that the resonance wavelength of the sensor initially locates at 1541.3 nm. Then the signal reaches a stable value of ~1542.5 nm when the sensor is exposed to the fixed methane concentration. The response time *T_res_* (defined as the time required for a sensor to reach 90% of the signal change) of the sensor was measured to be 50 s, which is comparable to that of the traditional LPFG gas sensor. However, the recovery time *T_rec_* (the time needed for a sensor to achieve 90% of the corresponding signal change for the desorption of methane) was nearly 65 s. In addition, the signal can recover to the initial resonance wavelength with a minimum standard deviation of less than 0.05 nm, which demonstrates that the graphene-based LPFG SPR sensor possesses good recoverability.

Finally, the reusability of the graphene-based LPFG SPR sensor is investigated. The sensor was alternatively exposed to methane gas with different concentrations. The signal was collected every 30 s, and the extracted resonance wavelength is plotted in Figure 7b. Clearly, the resonance wavelength increases and decreases as the sensor is exposed to methane gas with different concentrations. The final injection of nitrogen makes the resonant wavelength go back to its initial position. Meanwhile, the fluctuation of the initial resonant wavelength after several cycles is only 0.05 nm, indicating the excellent reusability of the graphene-based LPFG SPR sensor.

### 4.4. Finite Element Simulation

**Figure 8:**
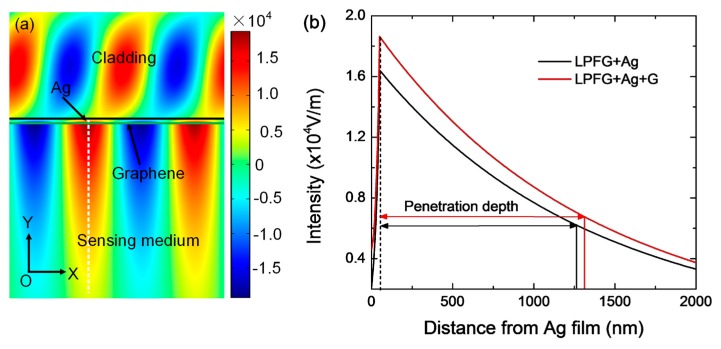
(**a**) Electric field distribution of graphene-based LPFG SPR sensor at the resonant wavelength of 1541 nm; (**b**) Cross-section plot of the electric field intensity along the direction perpendicular to the sensing interface.

To explain the sensitivity enhancement mechanism due to the presence of graphene, a physical model is built using the finite element analysis (FEA) method employing COMSOL Multiphysics. In the model, the dielectric constant of Ag (50 nm) comes from the handbook of Palik [28], the refractive index of the fiber cladding is 1.42, and the surface conductivity of graphene is modeled as σ_0_ = *e*^2^/4*ћ* in the considered spectral region [29], where *e* is the elementary charge and *ħ* is the reduced Planck’s constant. The calculated electric field mode profile of the grapheme-based LPFG SPR sensor is displayed in Figure 8a. One can see that a highly confined mixed SPR-FC hybrid mode is generated with the electric field intensity of 1.8 × 10^4^ V/m, which significantly enhances the light-matter interaction. To further quantitatively estimate the penetration characteristics of the mixed SPR-FC hybrid mode, the electric field intensity along the Y-direction (white dashed line) which is perpendicular to the sensing interface is extracted from Figure 8a, and compared with that of the Ag-coated LPFG SPR sensor in Figure 8b. Obviously, the electric field generated by the Ag/graphene structure is stronger than that of the structure without graphene and the electric field exponentially decays into the sensing medium. The penetration depth is defined as the distance where the electric field intensity decreases to 1/*e*. For the Ag/graphene structure, the penetration depth of the electric field into the sensing medium is 1260 nm, which is larger than that of the structures without graphene (1210 nm). Thus, the introduction of graphene can enhance the intensity of the electric field surrounding the sensing layer and enables the sensor to be more sensitive to the change in the gas concentration.

## 5. Conclusions

In summary, we proposed a graphene-based LPFG SPR sensor combining the advantages of LPFG and graphene. The experimental results show that the sensitivity of the graphene-based LPFG SPR sensor to changes in the concentration of methane gas is 0.344 nm%^−1^, which is improved 2.96 and 1.31 times with respect to the traditional LPFG sensor and the Ag-coated LPFG SPR sensor, respectively. Furthermore, the graphene-based LPFG SPR sensor exhibits good response characteristics and repeatability. Such a SPR sensing scheme points to a promising future for the graphene-based gas sensor with high sensitivity.
